# In this month’s Bulletin

**DOI:** 10.2471/BLT.25.000425

**Published:** 2025-04-01

In the editorial section, Ejemai Eboreime et al. (230) draw attention to the intersecting public health issues of climate, conflict and displacement in the Sahel. Nicole Bergen et al. (231) explain why monitoring of health inequalities is continuously needed to improve health equity.

Shanna H Swann talks to Gary Humphreys (234–235) about the health impact of exposure to endocrine-disrupting chemicals in the environment.

## China 

### Prevention of rabies 

Qi Yu et al. (247–254) evaluate elimination efforts.

## European Union, United Kingdom of Great Britain and Northern Ireland, United States of America 

### Which kits cost the most to ship?

Marie-Louise Wöhrle et al. (236–246) weigh components of lateral flow assays.

## India

### Improving postnatal outcomes

Jamie Sewan Johnston et al. (255–265) do a randomized controlled trial of mobile messages to families.

## Mali

### Implementing cardiovascular disease management

Issiaka Camara et al. (275–280) assess site readiness in three hospitals.

## Peru

### Equitable roll-out of new vaccines

Larissa A N Silva et al. (266–274) dissect trends in coverage

## Global 

### Capacity building for guideline development

Holger J Schünemann et al. (281–284) introduce an international training and certification programme.

